# Augmented Consulting: the future of primary care?

**DOI:** 10.3399/BJGPO.2020.0177

**Published:** 2021-03-03

**Authors:** Stuart Stewart, Yvette Pyne, Brian McMillan

**Affiliations:** 1 Centre for Primary Care and Health Services Research, Division of Population Health, Health Services Research & Primary Care, The University of Manchester, Manchester, UK; 2 Department of Population Health Sciences, Centre for Academic Primary Care, Bristol Medical School, University of Bristol, Bristol, UK

**Keywords:** Consultation skills, Family medicine, Information technology, Artificial Intelligence

## Introduction

Changes in clinical practice shaped by COVID-19 have included rapid and widespread adoption of digital technologies^1^ that, while enabling primary care physicians to continue to deliver health care remotely, have also created further barriers to the human connection so vital in medicine. Even before the pandemic, the physician’s use of the computer and electronic health record (EHR) had come to dominate the consultation in primary care.^2^ Notwithstanding the obvious utility of providing physicians with instant access to extensive patient information, and evidence-based guidelines, this uninvited third party in the consultation has disrupted the relationship between patient and doctor leading to poorer patient care^3^ and physician burnout.^4^

With global organisations now encouraging digital healthcare strategies,^5^ more technology is not only being invited into healthcare, but lauded as central to its delivery. Given the prominence of the computer within the consultation, it is entirely possible that more technology may drive a wedge further between the patient and doctor. Despite primary care rapidly embracing technology such as digitally delivered prescriptions, ‘fit notes’, and video consultations, recent patient-centred research articulates the experience through the patient’s eyes, balancing this enthusiasm.^6^ We are at an inflection point in healthcare’s digital transformation and it is vital that we drive the discussion around when and where technology is helping or hindering us in serving our patients. As purveyors of change, primary care physicians must take a lead in defining how digital technologies are used within the consultation. We recognise that the pace of technological change has been painfully slow and often disappointing, and many primary care physicians may oscillate between anxiety and apathy when thinking about digital solutions.

## Augmented Consulting

In this article, we present our vision of a fictional patient’s healthcare journey, bringing to life their experience through our own concept — Augmented Consulting (AC). Inspired by augmented reality, where technology is utilised in real time to augment and enhance real world experience,^7^ we define this concept as the selective utilisation of a range of digital technologies in unobtrusive and ambient ways to enhance the capabilities of doctors in caring for patients. What’s more, we take inspiration and draw on examples from research, health care, and other industries that demonstrate this ‘future’ vision is already a reality for some.

Let us consider ‘Sara‘, a fictional patient who wishes to consult with her family doctor about acute back pain. We start in her home where she uses accessible and asynchronous consultation tools to begin to tell her story in her own time and words, eliminating the constraints of 10-minute appointments and blurring the boundary of when the consultation begins. Intelligent triage systems will use patient-captured information, along with existing medical information in the EHR,^8^ and a signature of physical and psychological needs to arrange a consultation through an appropriate channel (such as, face-to-face, telephone, video, or e-consultion), and of an appropriate urgency and length with the right clinician. Intelligent triage systems will schedule appointments based on clinical priority, a departure from the potentially unsafe, yet widely used, first-come-first-served method. For Sara, an urgent face-to-face appointment is scheduled with her doctor. Pre-appointment consultation notes allow her doctor to prepare for the consultation, and serve as a reference point for other clinicians, reducing duplication and its associated inefficiencies as Sara does not have to repeatedly share the same information.

From the start of the consultation, Sara’s doctor is able to wholly concentrate on her as the EHR consultation notes are intelligently created through natural language processing technology similarly used in customer care.^9^ This system, far from being a passive documentation tool that simply transcribes the consultation, will identify critical keywords such as red flags and the patient agenda, along with recognising patterns of speech indicating conditions like bipolar affective disorder.^10^ Complementing speech analysis, facial recognition software already used to detect driver fatigue in cars^11^ could look for non-verbal signals to assess her outwardly displayed emotions and detect potentially contradictory micro-expressions, teasing apart what Sara is saying from how she feels. This would supplement the doctor’s own assessment of Sara’s emotional state while facilitating discussions around the psychological impact of her condition.

All of these data will be assimilated with existing EHR data and objective data from Sara’s wearable sensors and mHealth apps to better understand her condition in a far wider context. Furthermore, these streams of data will be assimilated into ‘nodes’ of information and simplified through data visualisation techniques, making abstract clinical information user-friendly for both Sara and her doctor, communicating a more understandable picture of Sara’s health. The system would present relevant information at exactly the right time to support, not supplant, clinical decision making, minimising interruptions to the patient–doctor exchange while preventing an over-reliance on the technology. In Sara’s case, a previous episode of anterior uveitis is flagged, which in the context of national back pain guidelines, highlights a potentially serious underlying diagnosis of Ankylosing Spondylitis, necessitating further investigations.

As Sara’s doctor begins to discuss a shared management plan, on-screen data visualisation can serve as a starting point for discussions, changing the relationship of the computer in the consultation from uninvited third party to welcome guest. After discussing the need for investigations, the conversation moves on to alleviating Sara’s symptoms. Truly individualised care is delivered through the integration of pharmacogenomic data within the EHR. Sara’s doctor avoids prescribing codeine for acute pain relief, opting for anti-inflammatory medication instead, as she has two inactive copies of the CYP2D6 gene, making her a poor metaboliser of codeine.^12^ Sara’s doctor offers a holistic plan, discussing an exercise regime, while incorporating the use of social prescribing integrated with local service listings, along with evidence-based mHealth apps validated to assist back pain management. At the end of the consultation, Sara has a digital record of the consultation and a reminder of the management plan, within her health records app empowering her to more fully engage in self-management of her own health.

## Conclusion

The EHR will be redefined and evolve into a truer reflection of the consultation, moving away from purely clinician-captured data into a dynamic record of subjective and objective data from both patient and clinician. Patient-captured information will offer novel insights into understanding the experience of illness through the patient’s eyes, opening up a wealth of opportunities for patient-centred research.

Beyond Sara and her back pain, machine learning (ML) will be crucial in assimilating these vast data throughout the consultation, while also offering novel insights into patterns of disease and disease management indiscernible to humans. Family medicine practices will collaborate with regional health informatics centres to develop and train ML algorithms on local datasets that represent local heterogeneity and provide solutions where guidelines that are based on healthy homogenous populations might not. At the heart of AC is an honest recognition and appreciation of the unique qualities of both humans and computers. The described technologies will not replace doctors but instead supplement their wide range of clinical and interpersonal skills with powerful and unfatigueable tools that provide specialist support and safety netting within consultations in real time. Additionally, these technologies may reduce the administrative burden and low value work in clinical practice, freeing up doctors’ time to connect with their patients.

The power of primary care as a patient-focused and continuous relationship, as opposed to the episodic and disease-focused nature of secondary care, is most clearly realised in this holistic technological approach. We believe improved patient–doctor relationships in primary care will arise from a greater (and more considered), not lesser use of technology. Through AC, digital technologies will truly become part of the consultation.
